# A comparison of four methods to define timing of acute kidney injury

**DOI:** 10.1186/cc9524

**Published:** 2011-03-11

**Authors:** KA Wlodzimirow, A Abu-Hanna, C Bouman

**Affiliations:** 1 Academic Medical Center, Amsterdam, the Netherlands

## Introduction

RIFLE provides standardized criteria for defining acute kidney injury (AKI) [1]. It is based on changes in serum creatinine (sCr), in relation to a premorbid sCr, and on urine output. When premorbid sCr is unknown, baseline sCr is estimated. Often only sCr is used (RIFLEcreat). Thus there are four methods for defining AKI: actual RIFLE, actual RIFLEcreat, estimated RIFLE and estimated RIFLEcreat. There is much interest for biomarkers predicting early AKI [2]. Critical for determining a biomarker's performance of AKI is the diagnosis of the first day of AKI (AKI-0). We compared the impact of four AKI definitions on determining AKI-0.

## Methods

An observational study for 6 months in ICU patients admitted ≥48 hours. For the first 7 days we calculated daily the number of patients diagnosed with AKI-0 using the four AKI definitions.

## Results

**Figure 1:**
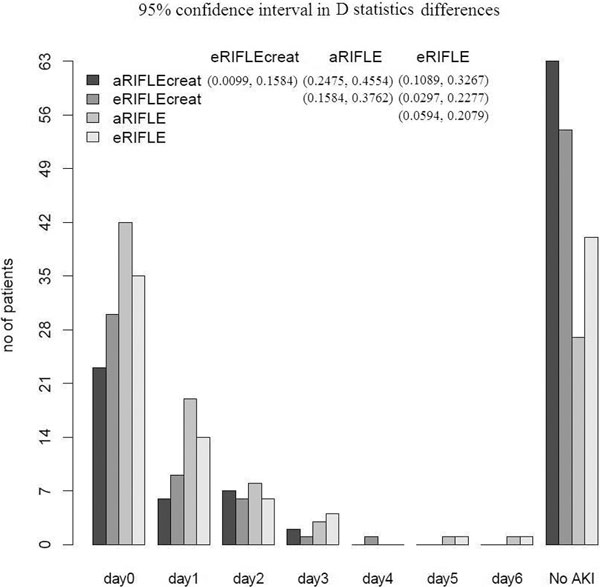
**Distribution of AKI-0**.

One hundred and one patients (39%) had a known premorbid sCr. Mean age and APACHE was respectively 64 (13) and 22 (7). Figure 1 (overleaf) shows the distribution of AKI-0.

## Conclusions

The early diagnosis of AKI is significantly reduced when urine output criteria are neglected in the RIFLE definition, and also when baseline sCr is estimated. This may significantly impact the assessment of biomarker performance.
